# A corpus for mining drug-related knowledge from Twitter chatter: Language models and their utilities

**DOI:** 10.1016/j.dib.2016.11.056

**Published:** 2016-11-23

**Authors:** Abeed Sarker, Graciela Gonzalez

**Affiliations:** Division of Informatics, Department of Biostatistics and Epidemiology, University of Pennsylvania, Philadelphia, PA 19104, United States

## Abstract

In this data article, we present to the data science, natural language processing and public heath communities an unlabeled corpus and a set of language models. We collected the data from Twitter using drug names as keywords, including their common misspelled forms. Using this data, which is rich in drug-related chatter, we developed language models to aid the development of data mining tools and methods in this domain. We generated several models that capture (i) distributed word representations and (ii) probabilities of n-gram sequences. The data set we are releasing consists of 267,215 Twitter posts made during the four-month period—November, 2014 to February, 2015. The posts mention over 250 drug-related keywords. The language models encapsulate semantic and sequential properties of the texts.

**Specifications Table**

**Table:** 

Subject area	*Biomedical informatics, data mining, natural language processing*
More specific subject area	*Social media mining for public health*
Type of data	*Text, Binary*
How data was acquired	*Drug-related chatter was directly collected from Twitter using the Twitter Streaming API. Posts were retrieved using drug names as keywords. To address the issue of common misspellings in social media, we employed a phonetic spelling variant generator that automatically generates common misspellings for the drug names.*
Data format	*Raw, processed*
Experimental factors	*Twitter posts were collected in raw format. Only basic preprocessing such as lowercasing was performed prior to the generation of the language models.*
Experimental features	*The language models were generated from the raw Twitter data after basic preprocessing. A neural network based technique is used to learn the distributed word representation models. The sequential language model is learned by computing probabilities of word n-gram sequences. The utilities of the two sets of models were verified via preliminary experiments of adverse drug reaction detection and text classification, respectively.*
Data source location	*N/A*
Data accessibility	*Data is within this article*

**Value of the data**

• The raw data containing drug-related chatter can be used to build prototype systems for a range of tasks in the domain of pharmacovigilance and toxicovigilance from social media, to assess user sentiments about the drugs, to estimate the effectiveness of distinct drugs and for other tasks important to the broader public health community.
• The distributed word representations were generated by varying multiple parameter combinations, thus ensuring that the different models capture different types of semantic information. Therefore, these models can be used for developing systems focused on mining knowledge associated with prescription medications.
• The n-gram language models capture sequential word occurrence probabilities and can be used for tasks such as text classification and text normalization.
• Python scripts are provided for downloading the tweets and for loading the two different sets of models.

## 1 Data

The data set consists of 267,215 Twitter posts, each of which contains at least one drug-related keyword. Two sets of language models accompany the raw data—the first is a set of models based on distributional semantics, which encapsulate semantic properties by representing word tokens as dense vectors, while the second set of models is based on n-gram sequences, capturing sequential patterns. All the data are available *via* our webpage, along with download/usage instructions: http://diego.asu.edu/Publications/Drugchatter.html. We will release more data and resources in the future via this link.

### 1.1 Characteristics of the data

**Fig. 1:**
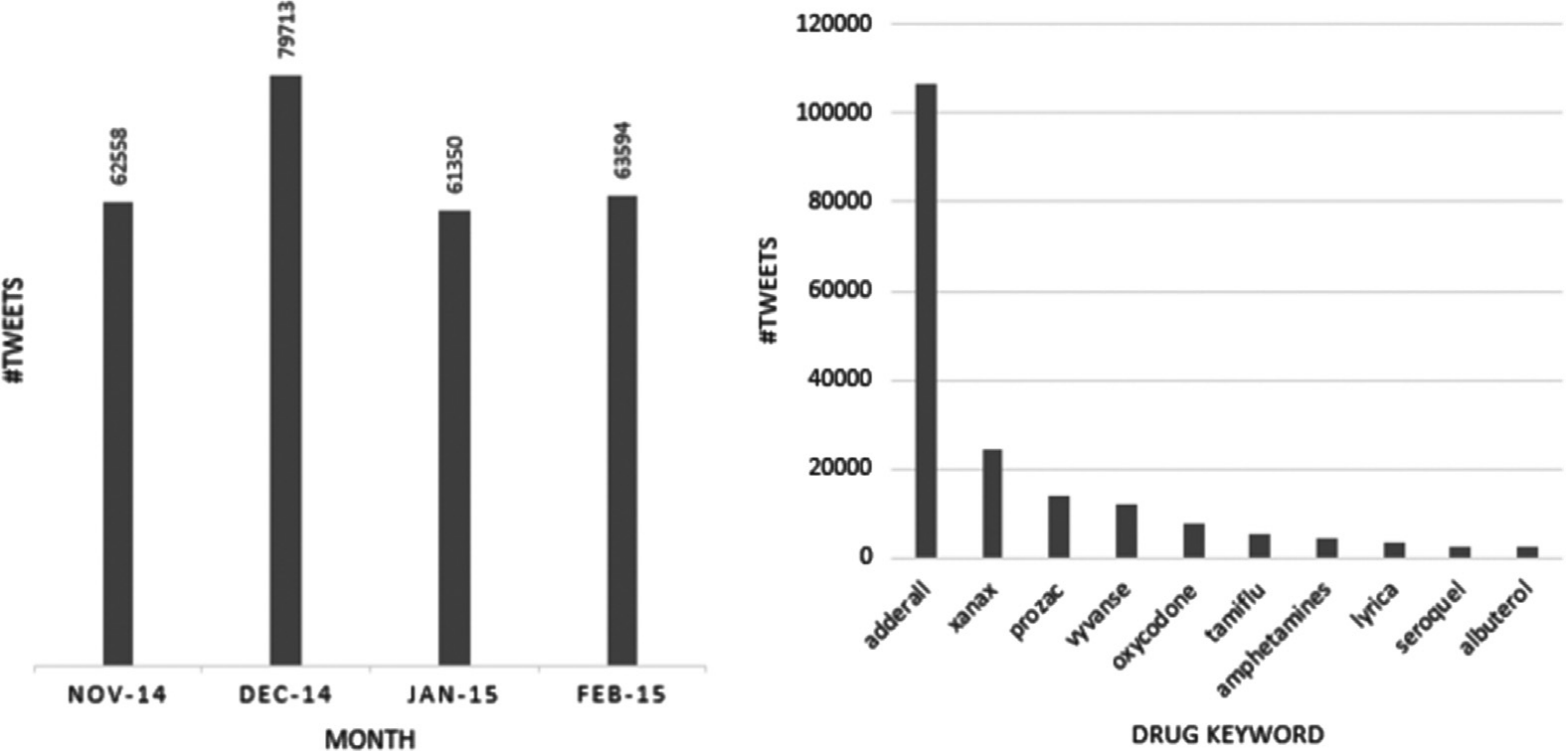
Left: distribution of tweets over four months, from November 2015 to end of February 2015. Right: Top 10 drug-related keywords on Twitter over the four-month period.

The posts were collected over a four-month period—November, 2014 to February, 2015 and they contain over 250 mentions of unique drug-related keywords. The monthly distribution of the data is shown in Fig. 1. The figure suggests that the numbers of drug-related tweets collected are fairly consistent over the four months, with December seeing the highest number of drug-related posts, perhaps because it is holiday season. Fig. 1 also presents the frequencies of the top 10 drug-related keywords in the data sample we are releasing. ‘*adderall*’ is by far the most frequently found drug-related keyword. Many other drug-related keywords are mentioned in the data set, presenting information about an assortment of classes of drugs such as antipsychotics, narcotics, stimulants, antidepressants, pain medications, sedatives and antivirals, to name a few.

## 2 Experimental design, materials and methods

### 2.1 Data collection

The data was collected from Twitter as part of a large-scale project on pharmacovigilance from social media [1]. To commence the collection of data, we first identified a set of drugs of interest. Our goal was to incorporate drugs used for a diverse set of conditions and those with high rates of usage. Therefore, in consultation with the pharmacology expert for the project, we focused on two criteria: drugs associated with chronic conditions and drugs with high prevalence of use. For the former criterion we initially chose drugs prescribed for conditions including but not limited to nicotine addiction, coronary vascular disease, depression, Alzheimer׳s disease, chronic obstructive pulmonary disease, hypertension, asthma, osteoporosis and type 2 diabetes. We later complemented our initial list with drugs prone to abuse, antivirals and biologics. For the second criterion regarding prevalence of use, we selected drugs from the IMS Health׳s top 100 drugs by sales volume for the years 2013, 2014 and 2015. Further descriptions of our drug selection process can be found in our past publication [2].

**Fig. 2:**
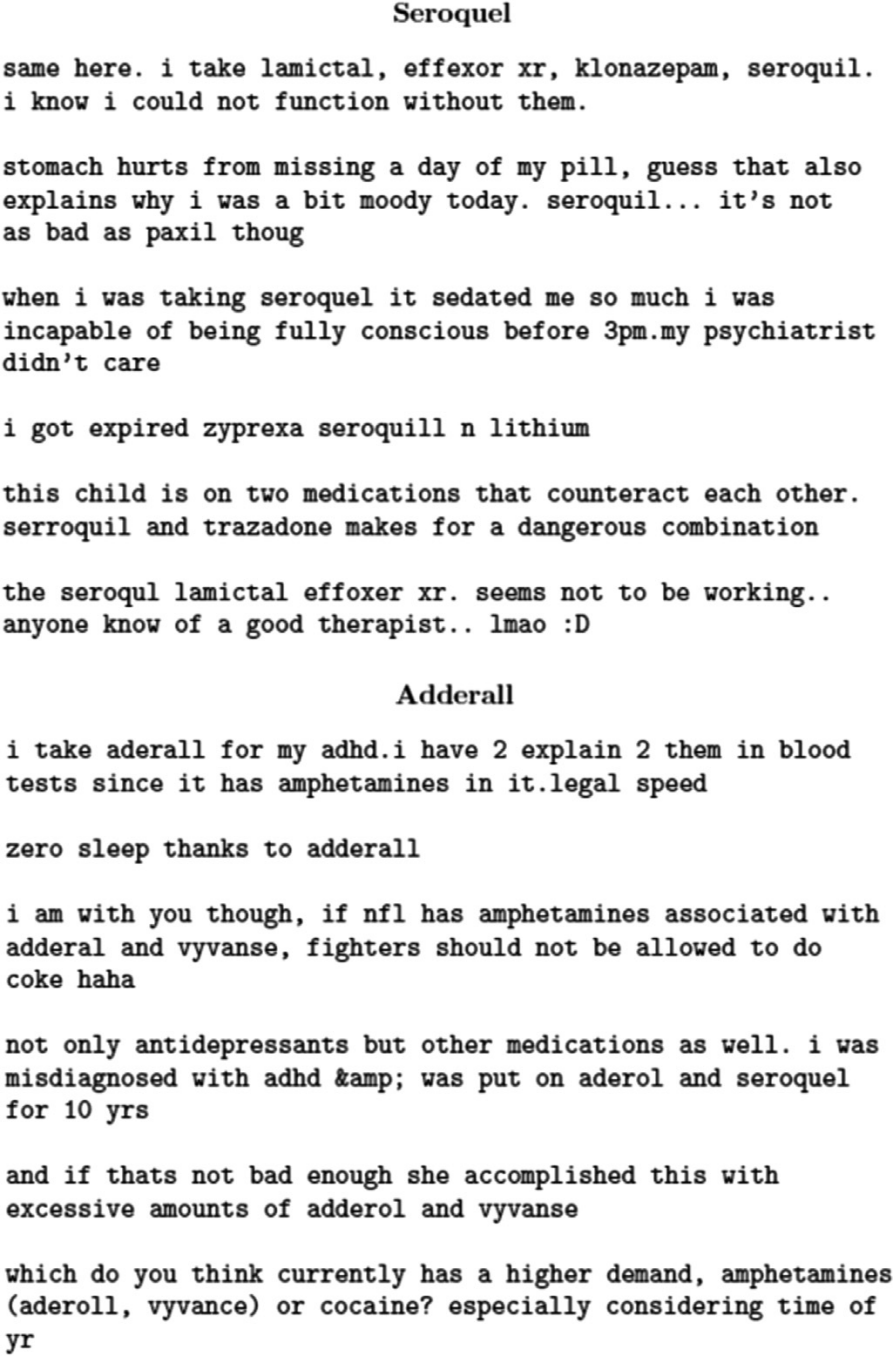
Sample tweets containing correct spellings and misspellings for the drugs Seroquel® and Adderall®.

**Fig. 3:**
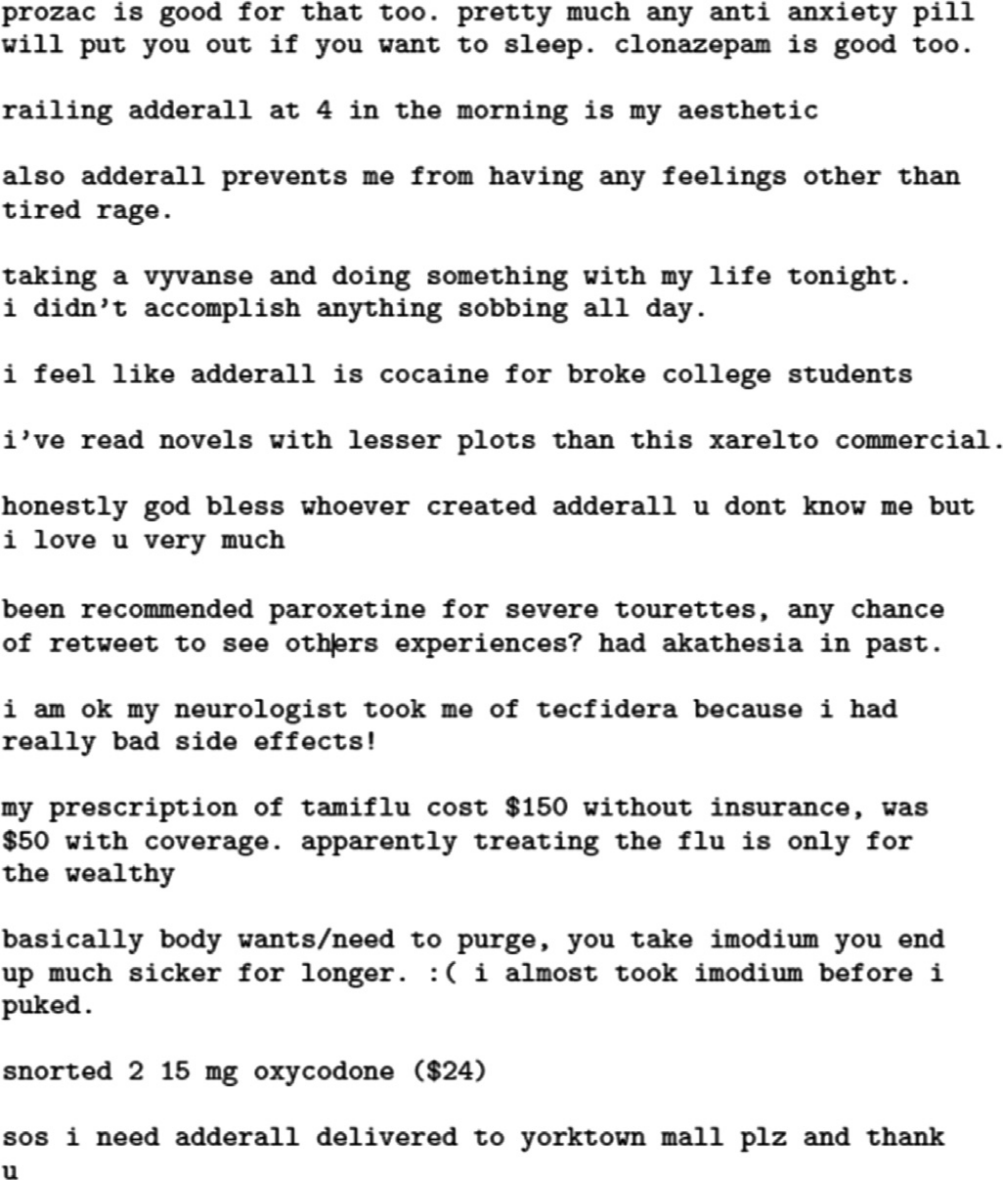
Sample tweets containing drug-related chatter. Collected between November 2014 and February 2015.

A major problem associated with collecting drug-related data from Twitter is that drug names are often wrongly spelled by the users. Fig. 2 shows tweets mentioning the two drugs Seroquel® and Adderall® *via* a variety of spellings, including the correct ones. As the figure suggests, using the correct spelling for a drug as the only keyword may fail to retrieve important tweets associated with that drug, leading to a lower recall than desired. Therefore, we used a misspelling generator [3] that, given the correct spelling for a drug, generates common phonetically similar misspellings for that drug. The misspelling generator runs in three steps. First, all lexical variants of a keyword within *one Levenshtein distance* (*i.e.,* difference of a single character insertion, deletion or substitution) are generated. Since these variants can be phonetically very dissimilar from the original keyword, a filter is applied which only keeps misspellings that have the same phonetic representations as the original keyword. Finally, to obtain a smaller set of misspellings, the Google custom search API is used to identify those that are frequently used as input to the search engine. The generator, which we have also made publicly available, can be used for other targeted data collection tasks from Twitter. For our work, we used the correct drug spellings and all the selected spelling variants, and the Twitter Streaming API to collect user posts. Fig. 3 presents a random sample of tweets associated with a number of drugs. The tweets appear to present a number of types of information about the drugs. Depending on the intent, distinct types of drug-related information can be mined from this data source, making it a valuable resource for the previously mentioned communities.

### 2.2 Language model based on distributional semantics

Using the word2vec^1^ tool, we prepared a number of phrase-level models. The word2vec tool is the current state-of-the-art in generating distributed word representations from natural language data. The algorithm applied by this tool constructs a vocabulary from an unlabeled data set and learns vector representations of the words by training a shallow neural network with two layers. Formally, given a set of terms t and their context windows w, the objective function is to set the parameters H that maximize P(w|t;H). The context, which is a symmetric window of terms around an input word, can be varied to modify properties of the distributed models (*e.g.*, varying window sizes may impact the capture of syntactic and semantic properties [4]). Similarly, the vector sizes and various other parameters influence the qualities of the models.

For the generation of the models, we varied several parameters including vector sizes and context window sizes. For the vector sizes, we generated models between the sizes 200 and 400. For the different vector sizes, we generated models using context windows within the range [2,9]. These models can be used to explore a variety of research tasks that can benefit from drug related knowledge generated directly from the users, such as exploring associations between drugs and adverse reactions, identifying drug abuse related signals, and, from a more NLP/data science perspective, the effects of different parameters such as context window and vector sizes in capturing semantic and syntactic properties. Such distributed word representation models are already being applied for research utilizing other sources of noisy health-related data, such as clinical reports [5] and such models have been generated from texts from other domains such as published literature [6] and generic social media [7]. However, perhaps due to the absence of available drug-related chatter data from social media, there are currently no such models available for this domain. We, therefore, believe that these models, along with the set of unlabeled data, will aid drug-related data mining tasks and will complement the resources generated from other domains (*e.g.*, clinical reports). For example, while clinical reports hold information discovered in clinical settings, social media chatter may hold information that are expressed by users in non-clinical settings. We present sample utilities of the models in the next section.

### 2.3 Language model based on n-gram sequences

We also generated sequential, n-gram language models. These language models capture the probabilities of n-gram sequences and such models have been applied in the past for tasks such as lexical normalization [8]. However, to the best of our knowledge, no such sequential probabilistic models are currently available for drug-related social media posts. Given a sequence of terms $w_{1}^{n}=w_{1}\ldots w_{n}$ the model learns n-gram sequence probabilities, such that approximations can be made for a sequence $w_{1}\ldots w_{m}$ *via* the expression $P\left(w_{1}^{m}\right)=\prod_{k=1}^{m}P\left(w_{k}|w_{k-1}\right)$. To generate the n-gram language models, we used the KenLM n-gram, language modeling tool [9]. We have made available a set of n-gram language models (*n*=2–4) from this data.

## 3 Utility of language models and data

We now briefly discuss two sample utilities of our two sets of models. Note that the qualitative results presented here were obtained using minimal settings, without any optimization.

### 3.1 Extracting adverse drug reaction signals using distributed word representations

We tested the possibility of utilizing our distributional semantic language models for exploring associations between drugs and adverse reactions. Past research has explored co-occurrence based techniques for identifying drug-adverse reaction associations. These early approaches primarily relied on pattern generation and detection [10] or handcrafted rules [11] for discovering drug-adverse reaction associations and are limited in multiple ways, particularly for unwieldy social media data, such as the ability to handle large volumes of text and discovering associations that do not follow specific rules. One of the properties of the distributional semantics model is the ability to capture semantic associations between terms based on co-occurrence in a large-sized corpus. Words/phrases learned by the models are represented as vectors in high-dimensional spaces, with contextually similar vectors appearing closer in semantic space. Therefore, we employed a simple cosine similarity measure to compare the *similarities* of drug keywords with sets of terms representing adverse reactions.

We chose three drugs for this experiment: two from past work on this problem—Trazodone and Aspirin [12]; while for the third drug, we randomly chose Xanax from the list of top 10 most common drug keywords from the data set, as shown in Fig. 1. Using one of our distributed representation models (vector size=400, context window size=9), we compared the cosine similarity values between a drug keyword and a set of adverse drug reaction terms. For simplicity, in the case of multi-word adverse reactions, we computed the average similarities for all the terms.^2^ In particular, we wanted to compare known adverse reaction representing keywords for each of the three drugs with adverse reactions that are not known to be associated with the drugs. We focused on non-serious adverse reactions, as we expected, based on recent research reports [13], that they occur more frequently in social media chatter.

**Fig. 4:**
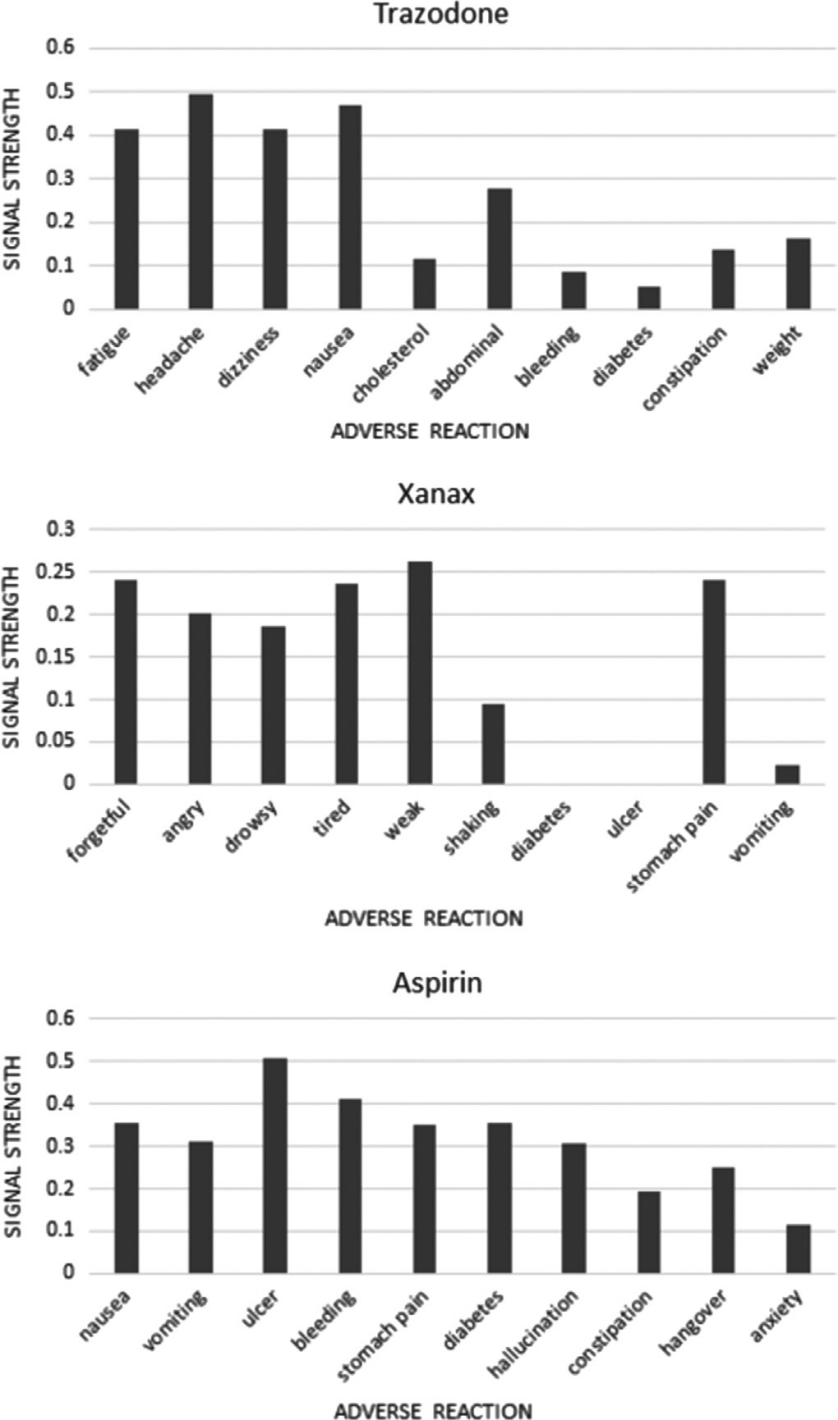
Drug-adverse reaction association signals for three drugs obtained using a distributed word representation model and cosine similarity.

Fig. 4 shows the cosine similarity distributions for the three chosen drugs and ten adverse reaction terms for each. For Trazodone, the first four adverse reaction terms (*fatigue*, *headache*, *dizziness*, *and nausea*) are known adverse reactions, as reported by [12], while the other six are randomly chosen adverse drug reactions not known to be associated with the drug. Even without any fine tuning, simple cosine similarity measures between the drug term vector and the adverse reactions clearly represent potential associations. For Aspirin, the five bars to the left in the figure represent known adverse reactions and the five to the right represent adverse reactions for which associations are not known. Although the associations are not as strong as in the case of Trazodone, there is a clear trend of lower values for the five associations to the right.

For Xanax, we chose a set of known adverse reactions and lesser known adverse reactions from Drugs.com. Fig. 4 illustrates similar findings for this drug as well, with strong signals for the five known adverse reaction terms to the left.

The results obtained from the distributional semantic model are very promising, specifically when considering the fact that these results are obtained without any pre/postprocessing of the data, the tuning of the signals, or any form of normalization of terms. We have also not explored how the window sizes and vector sizes affect the accuracies of these signals. We leave these exploration possibilities to the research community.

### 3.2 Sequential language models for text classification

The n-gram language models may also be applied to an array of tasks and since these models have been available for a long time, they have been utilized for lexical normalization [14], machine translation [15] and text classification [16], to name a few. We experimented with the possibility of utilizing our n-gram models for health-related text classification. All the tweets collected by us are intended to be related to prescription drugs. Thus, the tweets are a subset of all tweets that discuss health-related topics and it is likely that they will help identify a broader set of health-related tweets from a collection.

We first obtained a set of manually annotated health-related tweets from [17]. The data set contains 5128 tweets with about 35% of the tweets tagged as health-related. Our intent for this experiment was not to quantitatively analyze the effect of the language model in text classification. Instead, we focused on analyzing if these sequential models may be useful for the task. Given a sequence of tokens in a string, our model estimates the probability of that sequence based on the equation mentioned earlier. Because of the nature of our data, our intuition is that health-related tweets are likely to have higher probabilities based on this model. To test it, we chose a small sample of annotated tweets and computed scores for them using our n-gram model. We used a simple scoring mechanism for this small sample—we first generated absolute value scores for each sample tweet using the tetra-gram language model and then scaled the values to fit in the range [0,1].

**Fig. 5:**
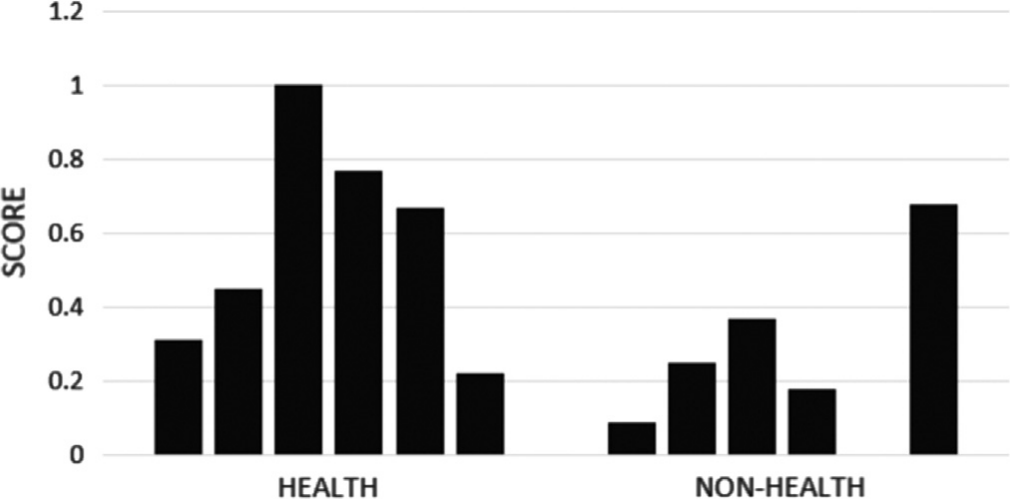
Sample tetra-gram language model scores for health and non-health tweets.

Fig. 5 presents a sample of probabilities obtained for a set of tweets from the health-related data set in [15]. Although we evaluated the impact of the language model in a small number of cases, the figure suggests that health-related tweets do tend to have higher scores based on our model. Such a feature can easily be integrated into text classification problems and we suspect it will improve performance.

### 3.3 Other utilities of data and language models

The previous subsections only present two sample utilities of the models among many. The task of mining prescription drug-related knowledge is currently of high interest and resources from several domains (*e.g.*, published literature, electronic health records and social media) are being utilized, including the combination of resources from multiple domains (*e.g.*, [18]). Unlike other domains, social media has emerged relatively recently as a domain of interest and the need for generating and making available social media based resources have been discussed in the recent literature [19]. Therefore, we believe that the unlabeled corpus and language models will be valuable to the research community.

As Fig. 2, Fig. 3 depict, drug-related chatter from social media can be used to mine knowledge about drug effectiveness, adverse reactions and misuse/abuse. Additionally, the data can be mined to obtain information about popular off-label uses of and user sentiments towards certain drugs. The data may also be used for relative comparison of two or more similar drugs. From the perspective of the data science and natural language processing communities, the data and the language models provide opportunities for developing and testing novel text mining algorithms. The distributional semantic models have been generated by varying important parameter values, so interested researchers can readily evaluate the performances of different parameter combinations for their chosen tasks. The models may also be used for generating visualizations of interesting components (*e.g.*, sentiments and adverse reactions) of drug-related chatter in semantic space *via* dimensionality reduction techniques, such as those presented in [20].

## Funding

This work was supported by National Institutes of Health (NIH) National Library of Medicine (NLM) Grant number NIH NLM 5R01LM011176. The content is solely the responsibility of the authors and does not necessarily represent the official views of the NLM or NIH.

## Footnotes

1 Available with the TensorFlow tool by Google: https://www.tensorflow.org/versions/r0.11/tutorials/word2vec/index.html. Accessed: 3rd November, 2016.

2 Note: composing vectors for multi-word expressions is an active research problem and these models may also be utilized to explore optimal techniques for composing multi-word adverse drug reaction vectors.
